# Telemedicine in interdisciplinary work practices: On an IT system that met the criteria for success set out by its sponsors, yet failed to become part of every-day clinical routines

**DOI:** 10.1186/1472-6947-8-47

**Published:** 2008-10-27

**Authors:** Antoinette de Bont, Roland Bal

**Affiliations:** 1 Department of Health Policy and Management, Erasmus University Medical Center, Post box 1738, 3000 DR Rotterdam, the Netherlands

## Abstract

**Background:**

Information systems can play a key role in care innovations including task redesign and shared care. Many demonstration projects have presented evidence of clinical and cost effectiveness and high levels of patient satisfaction. Yet these same projects often fail to become part of everyday clinical routines. The aim of the paper is to gain insight into a common paradox that a technology can meet the criteria for success set out at the start of the project yet fail to become part of everyday clinical routines.

**Methods:**

We evaluated a telecare service set up to reduce the workload of ophthalmologists. In this project, optometrists in 10 optical shops made digital images to detect patients with glaucoma which were further assessed by trained technicians in the hospital. Over a period of three years, we conducted interviews with the project team and the users about the workability of the system and its integration in practice. Beside the interviews, we analyzed record data to measure the quality of the images. We compared the qualitative accounts with these measurements.

**Results:**

According to our measurements, the quality of the images was at least satisfactory in 90% of the cases, i.e. the images could be used to screen the patients – reducing the workload of the ophthalmologist considerably. However, both the ophthalmologist and the optometrists became increasingly dissatisfied respectively with the perceived quality of the pictures and the perceived workload.

Through a detailed analysis of how the professionals discussed the quality of the pictures, we re-constructed how the notion of quality of the images and being a good professional were constructed and linked. The IT system transformed into a quality system and, at the same time, transformed the notions of being a good professional. While a continuous dialogue about the quality of the pictures became an emblem for the quality of care, this dialogue was hindered by the system and the way the care process was structured.

**Conclusion:**

To conceptualize what telemedicine does in interdisciplinary work practices, a fine-tuned analysis is needed to assess how IT systems re-shape the social relations between professional groups. Such transformations should not be exclusively attributed to the technology itself or to the professionals working with it. Instead we need to assess these technologies through an empirically grounded study of the sociotechnical functioning of telemedicine.

## Background

Information systems can play a key role in care innovations including task redesign and shared care [1-4]. In the Netherlands, information systems, such as digital diagnostic devices, databases and shared electronic patient records have been introduced to re-organize eye care [5,6], and to share tasks between ophthalmologists and optometrists.

Optometrists are a relatively new profession in the Netherlands. Optometric training programs at a bachelor level started only in 1990 and optometrists were first included in the health care system as paramedical profession in 2000 [7]. By comparison, in Great Britain optometrists were included into the National Health Service in 1948. The tasks performed by British optometrists, such as prescribing drugs or doing surgery, are well in the medical domain. Yet, Dutch optometrists are restricted to examining refraction faults and supplying contact lenses in most cases. Although they are allowed to perform medical tasks, such as using diagnostic drugs, they hardly ever do so in practice.

In the project we evaluated, telemedicine was introduced to delegate the collection of data from medical specialists to optometrists outside the hospital. In this project ten optometrists working in retail optician stores made digital images which were further assessed by trained technicians in the hospital. These technicians work closely with ophthalmologist and are trained by these doctors at the perimetry department to do certain diagnostic tests; they have no formal training outside the hospital. They are technicians in the field of ophthalmology, and not IT technicians which the notion 'technicians' might imply. Based upon the digital images made by the optometrists, the technicians in the hospital recommend whether a patient needs to consult the ophthalmologist.

Many demonstration projects in telemedicine have presented evidence of clinical and cost effectiveness and high levels of patient satisfaction. Yet these same projects often fail to become part of everyday clinical routines [8,9]. In this paper we would like to gain insight into the common paradox that an IT system can meet the criteria set out by its designers, sponsors, and evaluators, yet fail in the eyes of its users.

## Methods

In our study, we examined the development of a telehealthcare project over a period of three years. The aim of the project is to find cases in the population at risk for glaucoma. Glaucoma is an eye disease related to high intraocular pressure, which can lead to blindness.

In the Netherlands, people at risk for glaucoma are referred by the primary care physician to the ophthalmologist for tests and a physical examination. Because many people do not know they are at risk, case finding is suboptimal. In this project, clients of optical shops were offered an eye examination by an optometrist. Ten trained optometrists used a digital diagnostic technique (a nerve fiber analyzer, the GDx) to test the condition of the eyes. The nerve fiber analyzer produces an image and estimates the thickness of the nerve fiber layer using polarized laser light. The images are saved on the Internet in a database that is also accessible to the ophthalmologist and two technicians at the hospital. The optometrists also record data about the clients which the technicians need to detect new cases, such as the risk factors age, family history of glaucoma and high intraocular pressure. The electronic form offers them an opportunity to give some qualitative comments, like "could not make better pictures". The technicians, who are experienced GDx users, assess the images and decide whether additional testing at the hospital is necessary. They inform the optometrists by writing their assessment, their decision and their additional comments – e.g. "indeed very bad pictures" – in the database. The database thus enables sharing data and feedback between the optometrists and the hospital in a structured way and without much consultation.

We used a mixture of methods in our study [8,10-15]. One part of the study focused on the quality assessment of the digital images. Trained technicians examined the quality of the pictures to determine whether the optometrists were able to make digital pictures of sufficient quality. The judgment was based upon the centering, the focus and the exposure of the image. We analyzed the quality of the images of 1729 patients. At the time of the study, 2329 patients were screened. Due to technical problems which hindered electronic data collection at the start of the study, we excluded the first 500 patients. The data could be derived from the internet database which was filled during the study (see [16] for more a more detailed description of the methods used).

The other part of our study concentrated on the interpretations of the quality measurements of the pictures. We conducted 23 formal semi-structured or informal interviews with the project manager, the ophthalmologist, the two technical assistants and all optometrists involved (see Table 1). All participants (1 ophthalmologist, 1 project leader, 2 technicians and 10 optometrists) were interviewed at least one time. In this respect no selections were made. Some key informants, however, were interviewed several times. The ophthalmologist, the technicians and the project leader were interviewed in autumn 2001, spring 2002 and spring 2003. We also interviewed the optometrists yearly, but made different selections every year. In the first year we randomly selected four optometrists, in the second year, we interviewed them all and in the third year we selected the optometrists that did not meet the criteria of the technicians. In the second and third round of interviews, we used the quantitative data as input for qualitative methods [15].

The interviews were audio taped, transcribed (around 250 pages of verbatim transcripts) and analyzed. The informants' views regarding the quality of the pictures, the workload and their mutual relationship were identified and compared.

## The quality of the images

The ophthalmologist had to assure that certain quality standards were applied [17], before he could allow the optometrists to perform diagnostic tests. To do so, images of all the patients were assessed by both the optometrists and trained technicians. Consequently, the quality of the images was and increasingly became an important issue in the project. In the first interview at the start of the project in 2001, the ophthalmologist expressed his expectations about the quality of the pictures that the optometrists had to make. According to him it was easy to learn to make good pictures. 'In two weeks', he explained, 'you will learn to make these pictures'.

**Figure 1:**
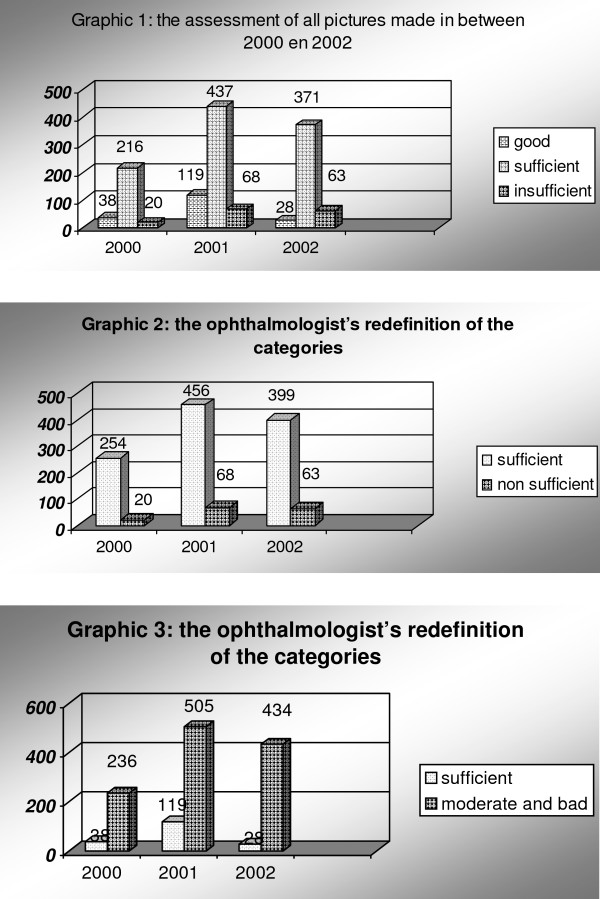
Ophthalmologists re-definitions

In 2002, the first quantitative data about the quality of the pictures became available. As we explained in the methods section, trained technicians examined the quality of the pictures during the screening process. According to these data 11% of the images were of poor quality. Most images were of sufficient (76%) or good (13%) quality (see figure 1 Graphic 1)[16]. Delegating the data collection to optometrists outside the hospital, moreover, reduced the workload of the hospital considerably. Only 27% of the patients seen by the optometrists were called for additional testing at the hospital department. A third of them (11% of all patients) consulted the ophthalmologist; the others were seen by the technicians only and were not referred to the ophthalmologist for further consultation. As we explained in the introduction the case finding was suboptimal before the introduction of the telecare service. Thanks to the telecare service, more cases could be detected. Yet, the ophthalmologist did not need to see all patients anymore. So, more patients were screened, but a smaller part of this group had to be seen by the ophthalmologist. At this point of the project, it was concluded that the project was a success.

We presented the data during a project meeting in November 2002. Focusing upon the percentage of the pictures that were actually used to screen patients, we concluded that 89% of the pictures met the quality standards. We explained that 11% of the pictures had not been used. In these cases, the hospital technician had asked the optometrists to make new images or asked them to send the patients directly to the hospital. This last group of patients was screened without the use of telemedicine. In other words, we redefined the categories 'sufficient and 'good' into 'sufficient', meaning that they could be used for diagnostic purposes, thereby redrawing the original presentation of the data, see figure 1 graphic 1, into something that looks like see figure 1 graphic 2.

The ophthalmologist, however, interpreted the data somewhat differently. According to his interpretation of the data only 10% of the pictures were of sufficient quality. In 90% of the cases the centering, the focus or the exposure of the image was not good. The technicians classified only 10% of the pictures as 'good'. Thereby he redefined the categories 'non sufficient' and 'sufficient' into 'moderate/bad', meaning that they did not live up to a high standard of image quality, thereby redrawing the original presentation of the data, see figure 1 Graphic 1, into something that looks like figure 1, Graphic 3.

As noted before in the literature, image quality is not a property of images themselves, but dependent on the ways in which images form a part of social and technical interactions and use [18]. This situated character of image quality was also apparent in the project. Whereas we as evaluators defined image quality as grossly sufficient 'for all practical purposes', that is, for the purpose of classifying patients to be or not be referred to the eye hospital for further consultation, the ophthalmologist rather took an ideal technical definition of image quality, reclassifying most of them as 'moderate/bad'. Against our pragmatic definition of image quality the ophthalmologist classified image quality against what was, in his experience, reachable using the GDx. In an interview in 2002, the ophthalmologist explained why he was dissatisfied with the quality of the pictures, relating this to the professional status of optometrist. He expected the optometrists to show more dedication in making better pictures. 'The quality of the images for screening is a problem. Some of the operators are satisfied too quickly. The quality differs per optometrist, also within this project. You cannot blame someone for a lack of experience, but you can blame him or her for a lack of dedication. An optometrist who does not show up for meetings reflects a lack of dedication, I think. He makes very bad pictures. He gets feedback but does not ask how to improve things' (Ophthalmologist, 2002). For the ophthalmologist the fact that optometrists did not seem to learn during the project became an icon for their lack of a professional attitude, which in turn led to a more negative evaluation of the quality of pictures. So, rather than just saying 'the glass is half empty', the qualification of image quality for the ophthalmologist became tied to standards of professionalism the optometrists did not live up to. It was not just their inability to make good pictures, but their lack of professionalism that was a reason to label even the large share of pictures that were actually used for diagnostic purposes as 'bad'.

Given that feedback became an important issue for the ophthalmologist as it showed professional attitude, we asked the optometrists in the third interview round about the way they were given and used feedback on image quality. Most optometrists said that they checked the comments the technicians made in the database. They said they retrieved the pictures to compare them with the comments, trying to understand why the picture in question was bad according to the hospital. Most also stored the feedback, after they checked it, in the records next to the pictures. We explicitly asked the optometrists whether they had considered asking for more feedback. Most of them never considered calling the hospital to ask for further feedback or explanation. 'I never call' (Optometrist 2, 2003), or 'I only see the ophthalmologist at the project meeting', and 'I don't have time to ask feedback about these pictures' (Optometrist 1, 2003). With the exception of one optometrist, no one asked the ophthalmologist how their 'bad' pictures could be improved.

In fact, it can be argued that the database offered few opportunities for the optometrists to respond to the feedback they received. Because of the structure of the database as a system to share data in a structured way without much consultation, asking for feedback was not an obvious course of action. They worked together – but at different locations and without having regular meetings. Because all communication went via the database, making a phone call to the technicians or the ophthalmologist – who did not even see the pictures himself – would have been unusual. Furthermore, because of a lack of informal communication there were few opportunities to correct for misinterpretations of the feedback that had been given. While both the ophthalmologist and the optometrists made a strong link between monitoring, quality and feedback, the use of the feedback by each was not visible to the other. Although feedback through the database was an integral part of the project, there were no mechanisms through which the reception and uptake of feedback could be made visible.

As a result, the optometrists became more and more dissatisfied during the project. According to the optometrists, they did a lot of work to make the best possible pictures, arguing they made more images than the hospital asked for and even asking patients to come back the following week to make better images. All the work put into making the best possible pictures for those patients, they perceived, was not taken into account. 'The assessment criteria are very high and have been raised during the project: a picture is never of good quality' (Optometrist 1, 2003). 'Why should we make these pictures? It just means extra work for us' (Optometrist 1, 2003).

The optometrists also said they were not given the opportunity to explain why the images were not better then they were, even after putting in the extra effort. They realized some images were not good when they sent them to the hospital, but they knew that each was the best picture that could be made given the circumstances under which pictures necessarily were taken. Reasons why images where not always of good quality according to the optometrist were, for instance, that a patient could not sit still or a patient had another eye condition, such as a cataract, which complicated the screening. Referring a patient directly to the hospital was an option, but this option was hardly taken into account. Not only for the ophthalmologist, but also for the optometrists the quality of the pictures was their main concern in this project.

By that time the number of pictures the optometrists made per month dropped. 4 out of 10 of the optometrists even stopped participating the project in which they had been involved for more than three years, at the moment the care process proved to be more efficient and effective [16].

During our study, both the ophthalmologist and the optometrists became increasingly dissatisfied with the project – the ophthalmologist about quality of the pictures and the optometrists about the time they spent on making pictures of satisfactory quality. In 2003, just under half of the optometrists decided to stop taking part in the project. According to the interviews we did with those optometrists that left the project, the perceived workload was the main reason. Although the ophthalmologist would assure that certain quality standards were applied by referring to the quality measurement and was able to demonstrate the efficiency of this project by referring to the number of consultations [16], it remained a small demonstration project. The six remaining optometrist are still making pictures for the Eye hospital. All the other optometrists in the region are no longer involved. Patients who do not visit one of these six optometrists, directly consult an ophthalmologist. In other words, the project failed to become part of routine health care delivery.

## Discussion

The glaucoma project showed how a technology can meet the criteria for success set out at the start of the project, yet fails to become part of every-day clinical routines. The evaluation study presents evidence of the quality of the pictures and the efficient use of the scarce consultations with ophthalmologist. In this respect the project is a success. However, both the ophthalmologist and the optometrists became increasingly dissatisfied with the project – the ophthalmologist about the quality of the pictures sent to him by the optometrists through the database and the optometrists about the time spent on making pictures of satisfactory quality. As half of the optometrists decided to stop participating in the project in which they had been taking part in for more than three years, it remained a small demonstration project.

Through a detailed analysis of how the professionals discussed the quality of the pictures, we could explain this paradox. We re-constructed how the notion of quality of the images and being a good professional were constructed and linked. Image quality thus seems to be embedded within the social relations in the project rather than being a property of the images themselves; those social relations were themselves also structured by the technology. The information system, we showed, transformed into a system for the assessment of professional quality and at the same time transformed the notions of being a good professional. Being a good professional changed from being a professional who is able to make pictures of sufficient quality 'for all practical purposes' into being a professional who is dedicated to constantly improving the quality of his or her work, in this case the quality of pictures of the eye.

The fact that monitoring quality is qualified as a 'feedback loop' is not simply a matter of labeling: it has significant consequences for the professionals and the monitoring system. A continuous dialogue about the quality of the work became an emblem for quality within the glaucoma project. However, the structuring of the care process and the systems prevented such a dialogue. Not only were the monitoring data irrelevant for the continuation of the project, its precise materiality – the internet application – prevented a continuous dialogue about the quality of the work and that lack of a dialogue became an emblem for the lack of quality of both the pictures and the professionals who made them. Knowing how the information systems were reconstructed and how the evidence of the evaluation study was part of this construction, we were able to explain why the project was not continued as could have been expected on the basis of the assessment of the effectiveness of the project.

## Conclusion

From our data one might get a strong perception that the supposed task redesign was not accepted by the ophthalmologist and that, therefore, the position of optometrists in the Netherlands as a relatively new profession might have been made more central in our analyses. The dissatisfaction with the pictures seemed to be due to the perception of the changes in accountability, power and stature. We do however not want to attribute this failure to the professionals working with it. Neither do we want to attribute the problems of communication to a technology. Instead we conceptualized what telemedicine does in interdisciplinary work practices *without *exclusively attributing this transformation to either the technology itself or to the professionals working with it [1]. Through an empirically grounded study of the functioning of telemedicine in a shared-care project, we came to a more fine-tuned analysis for assessing these technologies. In this analysis, image quality, professionalism, and technologies are not seen as fixed entities that then 'relate' to one another in more or less productive ways, but are rather seen as emergent properties of integrated care projects. That is, these properties should be treated as *situated outcomes *of such projects.

## Competing interests

The authors declare that they have no competing interests.

## Authors' contributions

AB carried the ethnographic study. AB and RB analyzed the data together and wrote the paper. All authors read and approved the final manuscript.

## Pre-publication history

The pre-publication history for this paper can be accessed here:


